# Pre-clinical lung squamous cell carcinoma mouse models to identify novel biomarkers and therapeutic interventions

**DOI:** 10.3389/fonc.2023.1260411

**Published:** 2023-09-25

**Authors:** Priyanka Sahu, Chantal Donovan, Keshav Raj Paudel, Sophie Pickles, Vrushali Chimankar, Richard Y. Kim, Jay C. Horvart, Kamal Dua, Antonio Ieni, Francesco Nucera, Helle Bielefeldt-Ohmann, Sarah Mazilli, Gaetano Caramori, J. Guy Lyons, Philip M. Hansbro

**Affiliations:** ^1^ Immune Health, Hunter Medical Research Institute, University of Newcastle, Newcastle, NSW, Australia; ^2^ University of Technology Sydney, Faculty of Science, School of Life Sciences, Sydney, NSW, Australia; ^3^ Centre for Inflammation, Centenary Institute and University of Technology Sydney, Faculty of Science, School of Life Sciences, Sydney, NSW, Australia; ^4^ Discipline of Pharmacy, Graduate School of Health, University of Technology Sydney, Sydney, NSW, Australia; ^5^ Department of Human Pathology in Adult and Developmental Age “Gaetano Barresi”, Section of Anatomic Pathology, University of Messina, Messina, Italy; ^6^ Pneumologia, Dipartimento di Scienze Biomediche, Odontoiatriche e delle Immagini Morfologiche e Funzionali (BIOMORF), Università degli Studi di Messina, Messina, Italy; ^7^ Australian Infectious Diseases Research Centre, School of Chemistry and Molecular Biosciences, University of Queensland, St. Lucia, QLD, Australia; ^8^ Department of Medicine, Boston University School of Medicine, Boston, MA, United States; ^9^ Department of Dermatology, The University of Sydney at Royal Prince Alfred Hospital, Sydney, Australia, and Centenary Institute, The University of Sydney, Sydney, NSW, Australia

**Keywords:** lung cancer, squamous cell carcinoma, animal models, risk factors, genetic and epigenetic alterations

## Abstract

Primary lung carcinoma or lung cancer (LC) is classified into small-cell or non-small-cell (NSCLC) lung carcinoma. Lung squamous cell carcinoma (LSCC) is the second most common subtype of NSCLC responsible for 30% of all LCs, and its survival remains low with only 24% of patients living for five years or longer post-diagnosis primarily due to the advanced stage of tumors at the time of diagnosis. The pathogenesis of LSCC is still poorly understood and has hampered the development of effective diagnostics and therapies. This review highlights the known risk factors, genetic and epigenetic alterations, miRNA biomarkers linked to the development and diagnosis of LSCC and the lack of therapeutic strategies to target specifically LSCC. We will also discuss existing animal models of LSCC including carcinogen induced, transgenic and xenograft mouse models, and their advantages and limitations along with the chemopreventive studies and molecular studies conducted using them. The importance of developing new and improved mouse models will also be discussed that will provide further insights into the initiation and progression of LSCC, and enable the identification of new biomarkers and therapeutic targets.

## Introduction

Lung cancer (LC) is the leading cause of cancer death (11.6%), with an estimated 1.8 million deaths (18% of total cancer deaths) worldwide (1). Global age-standardised incidence rates of tracheal, bronchus, and lung cancer decreased by 7·4% in males but increased in females by 0·9%, 2010-19 (2). Although the survival rates of LC patients have improved in the last 10 years, over half of patients die within 1 year of diagnosis largely due to the majority being diagnosed with advanced stage disease, precluding treatment with curative intent (3). LC is classified as small-cell lung carcinoma, which accounts for <15% of LC cases, and non-small-cell lung carcinoma (NSCLC), which accounts for 85% of all reported LC (4). NSCLC is further divided into 3 main subtypes; lung adenocarcinoma (LUAD), Lung squamous cell carcinoma (LSCC), and large cell carcinoma. LUAD is the most common NSCLC and accounts for 40% of LC cases and occurs in the lung periphery whereas LSCC accounts for 30% of all the LC cases and mainly originates in the bronchial epithelium (5). The incidence of LC reported varies substantially worldwide, and in Asian countries, the incidence is affected by other secondary smoke exposure. Several findings may reflect the reason for the declining LSCC cases reported in Asian countries compared to western countries (6). LSCC was the most common histologic subtype amongst men prior to 1990s. In the developed and Industrialized nations, smoking rates peaked first in men, followed by women thus causing increased rates of LSCC than LUAD. LSCC incidence and mortality went up before declining following the initiation of comprehensive tobacco control program leading to declined LSCC rates in the US, Canada, many European countries, and Japan (7, 8). However, this was not the case in other countries such as Norway, Finland, Spain, and France where the LSCC cases are still stable (8). The incidence rates of LSCC on the Indian subcontinent and Taiwan has also declined gradually over recent years with an increased LUAD rates (9, 10). This decline in LSCC is attributed to high usage of smokeless tobacco. The rise of LUAD incidents compared to LSCC are greater in women and in overall population due to exposure to biofuels. Although they exhibit a lower incidence of LSCC but a higher mortality burden compared with developed countries due to unequal access to healthcare leading to delayed diagnosis and treatment, environmental contamination, and sociocultural barriers (6).

Cigarette smoke (CS) exposure is the major risk factor in the development of lung cancer, particularly LSCC as smoke injures the airway epithelium and can lead to pre-neoplastic changes that include hyperplasia (increased cell number), squamous metaplasia (expansion of squamous epithelium) and increasing grades of dysplasia (presence of abnormal cells) and ultimately carcinoma *in situ* (11). Whilst most pre-neoplastic lesions do not lead to the development of LSCC, a subset do progress on to invasive disease that is suggested to be in part attributed to breakdown in the to host immune surveillance (12, 13).

LSCC has lower 5-year survival rate largely due to detection at a late stage where therapeutic options are often ineffective (14). Despite the advancement of immunotherapies and regulatory approval of immune checkpoint inhibitors, such as PD-1 inhibitors, there remains an urgent need for additional and more effective treatment options for this important subset of LC patients. Here, we review current knowledge on LSCC pathogenesis, including risk factors, molecular alterations, current animal model, and highlighting the importance pre-clinical studies in the identification of biomarkers and novel therapies for improved diagnosis and treatment of patients.

### LSCC risk factors in humans: CS & chronic obstructive pulmonary disease (COPD)

**Figure 1:**
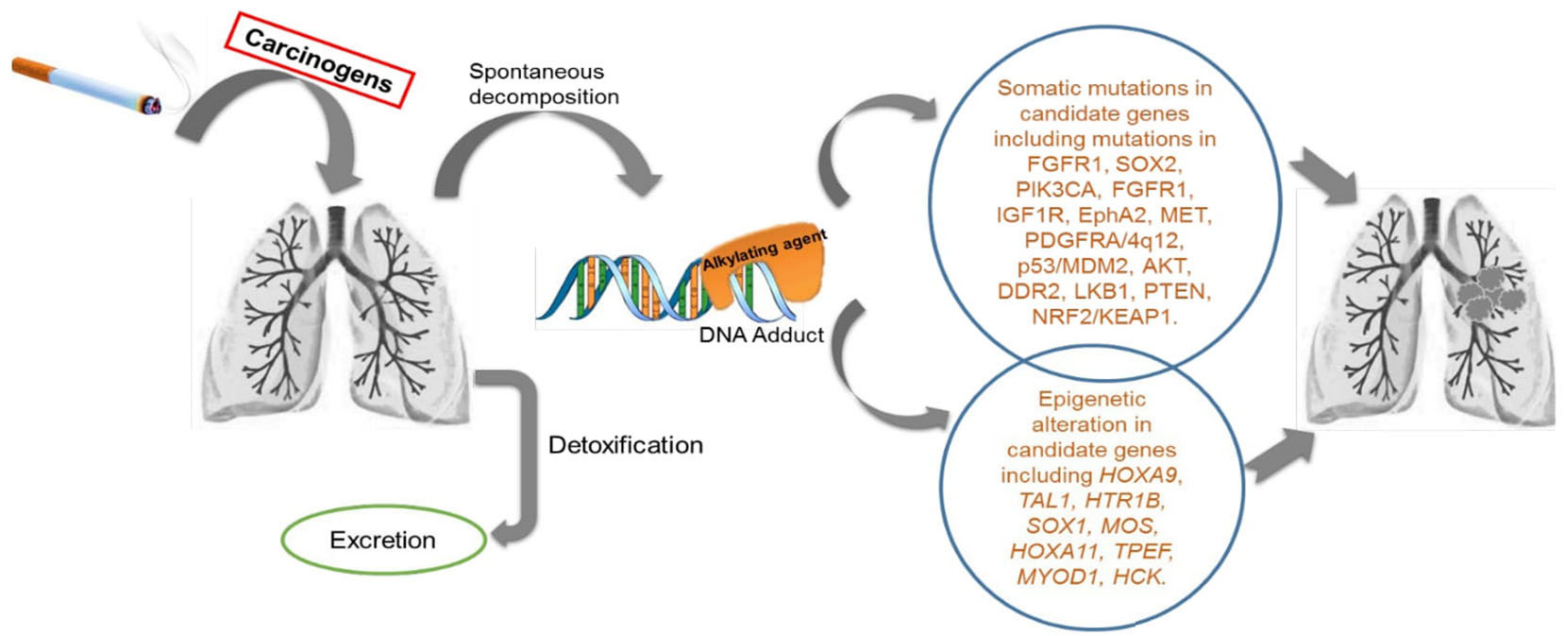
Schematic diagram of the activity of cigarette smoke exposure in human lungs: Cigarette smoke (CS) consists of more than 60 tumour initiating carcinogens tested in both laboratory animal models and in humans. There are 20 potentially known carcinogens found in a burning cigarette which are involved in lung carcinogenesis. These include polycyclic aromatic hydrocarbons (PAH), N- nitrosamines, 1,3-butadiene and ethylene oxide, cadmium, and the radioactive compound 210PO. Few of these compound needs to be metabolically activated or undergoes decomposition to exert the carcinogenic effect. There are detoxifying enzymes such as cytochrome P450, glutathione S-transferase and UDP-glucuronosyl transferases which helps in removal of metabolically activated carcinogens. DNA adducts are formed during this process. When these adducts are left unrepaired by the DNA repair enzymes in the body, they result in genetic changes in key genes for cellular function. These altered genes, eventually leads to carcinogenesis.

Extensive epidemiological data have clearly established CS exposure as a risk factor for LSCC, which is greater than for any other type of NSCLC (15–17). CS is comprised of >60 carcinogens, such as N-nitrosamines, polycyclic aromatic hydrocarbons, aldehydes, carbon monoxide, hydrogen cyanide, nitrogen oxides, benzene, toluene, phenols and other components (18–20). Some compounds require metabolic activation or spontaneous decomposition to have carcinogenic effects that lead to gene alterations and transformations (**Figure 1**) (18, 19). A pooled analysis of 13,169 cases and 16,010 controls revealed that LSCC was predominant in male heavy smokers compared to other groups, with an elevated odds ratio of 103.5 (95% Confidence Interval 74.8-143.2) (21). Current smoking men had higher risk of lung cancer than women irrespective of smoking quantity, duration or time since quitting (22). In addition to CS, other inspirable environmental pollutants/factors, such as bushfire/wildfire and solid fuel smoke, are also likely to be risk factors for LSCC development and warrant further research (23).

COPD is now the 3^rd^ commonest cause of death globally (24). It is a chronic inflammatory lung disease of the lower airways and parenchyma often induced by chronic CS exposure and is characterized in advanced stages by severe breathing difficulties (25–27). Oxidative stress, increases in inflammatory cytokines, and an imbalance between protease and anti-protease levels, predispose individuals to developing COPD (28). The key pathological features of COPD include chronic bronchitis, remodelling and narrowing of the small airways, destruction of the lung parenchyma and emphysema. This results in increased airflow resistance, reduced lung elastic recoil, small airway closure and gas trapping ultimately leading to impaired lung function and gas exchange (14). Significantly, COPD patients have four-fold greater risk of developing LSCC than the general population (17). Thirty percent of patients diagnosed with moderate or even mild COPD subsequently develop LSCC, suggesting that COPD is an important contributing factor in the development and progression of this squamous subtype (17). Furthermore, chronic inflammation in COPD can increase the expression of growth factors, such as epithelial growth factor (EGFR), and activates transcription factors, such as NF-κB, that favor lung tumorigenesis (29). Studies of airway epithelial cells have also identified single nucleotide polymorphisms in specific genes in airway epithelial cells, including hedgehog-interacting protein, A disintegrin and metalloproteinase 19 (ADAM19), family with sequence similarity 13 member A (FAM13A), and cholinergic nicotinic receptor locus (CHRNA), that occur in both COPD and LSCC (30, 31). These findings highlight the similarities between genetic mechanisms underlying the development of both diseases. Epigenetic changes such as DNA methylation, histone modifications, non-coding RNA regulation are found to be similar in COPD and LSCC (32). Thus, in addition to CS exposure, there are genetic and epigenetic change that are shared factors for COPD and LSCC suggesting CS associated COPD is a major risk factor for LSCC.

### Molecular drivers of human LSCC genomic alterations

Identifying the mutations responsible could lead to the development of effective targeted molecular therapies. A comprehensive characterization of genomic alterations in LSCC from a large cohort of 178 patients was conducted by The Cancer Genome Atlas (TCGA) that reported a mean of 360 exon mutations, 165 genomic rearrangements, and 323 segments of copy number alterations per tumor that defined the LSCC genomic landscape (33). TCGA reported recurrent mutations in 18 genes, including tumor protein p53 (*TP53)* mutations in 86% LSCCs. Mutations were also reported in oxidative stress genes including nuclear factor erythroid-2-related factor*-*2 (*NFE2L2*), cullin-3 (*CUL3*) and Kelch-like ECH-associated protein-1 (*KEAP1*) and genes associated with squamous differentiation including the amplification of tumor protein p63 (*TP63*) SRY (sex determining region Y)-box 2 (*SOX2*), and loss-of-function mutations in Notch homolog-1 and 2, (*NOTCH1* and *NOTCH2*) and Achaete-Scute family BHLH transcription factor-4 (*ASCL4*), and focal deletions in Forkhead box protein P1 (*FOXP1*) were also reported in 44% of cases. A recent study was conducted on 39 lesions from 29 patients with pre-invasive lung carcinomas using whole genome sequencing and comparing with TCGA (34). A wide range of similar mutation burden in genes such as *TP53*, *CDKN2A*, *SOX2*, *AKT2* and *NOTCH1* was noted between pre-invasive lesions and LSCC data in TCGA.

Although TCGA set a precedent for mutational profiling of LSCC a number of additional studies have also identified somatic alterations in LSCC (35). The catalytic subunit (p110α) of *PI3K*, *PI3KCA*, which plays a significant role in the PI3K-AKT pathway, has copy number gains in LSCCs (36–39). Hot-spot mutations in phosphatidylinositol-4,5-bisphosphate 3-kinase catalytic subunit-α (*PIK3CA)* and B-raf proto-oncogene, serine/threonine kinase *(BRAF*)^V600E^ promote LSCC development (40). Fibroblast growth factor receptor 1 (*FGFR1*) amplification is another key dysregulation in LSCCs and inhibition of *FGFR1* in mouse models and cell lines inhibits tumor growth (35, 41). Activation of the insulin-like growth factor-1 receptor *(IGF1R*) is involved in triggering pathways such as *RAS/RAF/MAPK*, which are important in cell proliferation. Over-expression of *IGF1R*, located on chromosome 15q26, is often observed in both LUAD and LSCC (42, 43). Over-expression of *EPHA2* is frequently observed in LSCCs but not other LCs (44). *MET* over-expression is associated with abnormal cell proliferation and invasion, which is common in LSCC compared to other NSCLCs (45, 46). Amplified *PDGFRA* is more frequent in LSCCs than ACs. An *in vitro* study with NCI-H1703, a LSCC cell line, showed that *PDGFRA* inhibition leads to anchorage-dependent cell growth, suggesting that *PDGFRA* is an oncogene in LSCC (47). *p53* mutations are very common and occur in 65% of LSCC and ~50% of NSCLCs (48). Around 75% of *p53* mutations are missense mutations and these often produce gain-of-function mutant p53 (35). p53 inactivation also results from over-expression of MDM2 that ubiquinates p53 and marks it for degradation (49), which is frequent in both LUAD and LSCC (50, 51). *DDR2* kinase gene mutations are reported in 9 out of 277 (~3%) of LSCC cases (52). Altered *PTEN* and *NRF2* expression is also observed in LSCC but not in LUAD (53, 54). p40 is a distinguishing marker in LSCC which differentiates it from LUAD (55). A recent comparative study concluded the overlapping and non-overlapping mutation burden between LAUD and LSCC. In this study, researchers identified 38 gene mutations in LAUD and 20 in LSCC using MutSig2CV. Out of these mutations, only 6 genes, *TP53*, *RB1*, *ARID1A*, *CDKN2A*, *PIK3CA*, and *NF1*, were observed to common in both tumor types although, the frequency of *TP53*, *CDKN2A*, and *PIK3CA* mutation was higher in LSCC (56).

Collectively, these studies show that different mutations may be common and/or unique to LSCC versus LUAD. These specific mutations may be novel targets for LSCC and represent key opportunities to develop LSCC-specific therapies in a landscape where many existing therapies for LUAD are ineffective for LSCC.

### Epigenetic alterations

The induction of carcinogenic processes can also be driven by the accumulation of epigenetic changes, which results in the dysregulation of key oncogenes, tumor-suppressor genes, and DNA repair or housekeeping genes. Epigenetic alterations, demethylation, hypermethylation and hypomethylation are as frequent as genomic mutations in LC (57) (**Table 1**). Some epigenetic alterations can delineate between LSCC and AC, for example demethylation in the promoter region of Cancer/Testis Antigens (CTA) expression is highly associated with LSCC but not LUAD (57). Hypermethylation of gene promoters are also associated with transcriptional silencing of tumor suppressor genes and it is thought that this promotes the initiation and progression of carcinogenesis. Hypermethylation typically occurs in CpG rich regions/islands at or near gene promoters and promoter hypermethylation may be a marker for early diagnosis, disease stage and predictor of patient outcomes in LSCC. Notably, the methylation pattern of the *CYTL1* promoter region changes between early and advanced stages of LSCC (57).

**Table 1:** Epigenetic alterations in squamous cell carcinoma: A detailed list of genes that are involved in epigenetic regulation in LSCC development.

Gene/s	Expression in LSCC	Epigenetic alteration	Reference
*MAGEA*	Higher expression of MAGE-A3/6 in males than females	Demethylation in P2-promoter region	(58)
*SBSN*	Upregulated in carcinoma-in-situ, growth promoter	Demethylation in P2-promoter region	(59)
*TKTL-1*	Over-expression in carcinoma-*in situ* and associated with reduced survival	Demethylation in P2-promoter region	(59, 60)
*ZNF711*	Upregulated	Demethylation in P2-promoter region	(57)
*G6PD*	Over expression	Demethylation in P2-promoter region	(57)
*TP73*	Over-expression of ΔNp73 mRNA	Hypomethylation in the promoter region	(61)
14-3-3	Increased expression *via* interaction with IGF-1	Hypomethylation in DNA induced transcriptional activation	(62)
*CCDC37, CYTL1, CDO1, SLIT2, LMO3*	Downregulated	Hypermethylated CpG site of promoter region	(63)
*SERPINB5*	Upregulated	Hypomethylation in CpG site of promoter region	(63)
*MGMT*	Upregulated	Aberrant methylation in promoter region linked to increased p53 mutation occurrence	(64–66)
*CDH1*	Downregulated	Aberrant methylation associated with poor prognosis	(65, 67)
*TIMP3, DAPK1*	Decreased transcription level	Hypermethylation of CpG islands	(68)
*RASSF1A*	Higher expression associated with improved survival	Hypermethylation	(69)
*SHOX2*	DNA methylation of SHOX2 distinguished between malignant and benign tumours	Hypermethylation	(70)
*CALCA, EVX2, GDNF, MTHFR, OPCML, TNFRSF25, TCF21* *PAX8, PTPRN2, PITX2*	Gene silencing associated with LSCC	Hypermethylation of CpG islands	(71, 72)

The activity and expression of individual gene is mentioned.

Hypermethylation of the *p16* and/or *O* ^6^-*methylguanine-DNA methyltransferase* gene promoters was detected in sputum samples from LSCC patients up to 3 years preceding or at the time of diagnosis (64), and is a potential biomarker for early detection (73). Hypermethylated *RASSF1A* is associated with early recurrence of LSCC and is considered an informative diagnostic biomarker for remission (65, 71). It is important to assess methylation profiles in tumor versus non-tumor tissue. In one study, the methylation profile of 42 gene loci were analyzed in a collection of 45 LSCCs and compared with non-tumor lung tissues from the same patients (72). *CALCA*, *EVX2*, *GDNF, MTHFR, OPCML, TNFRSF25, TCF21, PAX8, PTPRN2* and *PITX2* were hypermethylated in LSCC compared to non-tumor tissues (71, 72). Whilst many epigenetically modified genes have been identified in LSCC that differentiate it from other LCs, the specific roles of these altered genes in the development, progression and recurrence of LSCC are yet to be elucidated.

Histone modifications and chromatin remodelling are critical in the pathogenesis of NSCLC and other CS-induced lung diseases including COPD. CS-induces post-translational histone modifications in H3 and H4 in lung cells. These are potential biomarkers as they play a key role in the epigenetic state in the pathogenesis of CS-induced LSCC and NSCLC (74). A study of 408 NSCLC tissues assessed the global modification status of histone H3 and H4 and their association to tumor recurrence with LSCC patients having lower levels of H3K4 dimethylation (73). Li et al. showed that in NSCLC higher expression of H3K4 histone demethylases (KDM1A, KDM5A, KDM5B and KDM5D) was associated with poor overall survival, while patients with low expression of H3K4 histone methyltransferases SMYD3 had worse prognosis (75). Another study by Leng et al. assessed KDM6A, a member of the mixed-lineage-leukemia (MLL2) H3K4 methyltransferase complex, and reported that expression of KDM6A protein was higher in NSCLC tissues than in corresponding para-cancer tissues and that higher expression was associated with poor prognosis (76). It should be noted that these studies were of NSCLC but none of them were on LSCC in particular.

Although studies have established the genetic and certain epigenetic alterations in advance stage LSCC, future studies assessing genetics and epigenetics in the developing early pre-malignant lesions would be important to understand the pathophysiology of LSCC development.

### MicroRNAs (miRNAs) as potential biomarkers

LSCC antigen, neuron-specific enolase, and CYFRA 21-1 are reported biomarkers of NSCLC (74), however, they do not detect the early stages of these tumors and therefore early biomarkers are required. miRNAs are small non-coding RNAs that are emerging as reliable biomarkers for early diagnosis of respiratory diseases including LSCC (73, 75, 76). Thus, the identification of unique miRNAs or signatures would be a major advance in LSCC diagnosis. The miRNAs commonly identified in LSCC patients are summarised in **Table 2**. One study profiled miRNA expression in 61 LSCC compared to normal lung samples. A total of 15 differentially expressed miRNAs was identified, including increased expression of miR-17/92 clusters and paralogous miR-106a-363 and miR-93-106b, miR-182-183 clusters (84). When 474 human miRNAs were mapped onto the array comparative genomic hybridization database (a portal of databases with thorough data on human genome aberrations and variations), 77 were linked with DNA copy number changes in LSCC, which included 40 associated with amplified regions and 37 to regions deleted. In another study, mature miR-218 produced from two precursors hsa-mir-218-1; MI0000294 and hsa-mir-218-2; MI0000295, was downregulated in LSCC compared to normal lung tissue (77). An investigation of miRNA in Chinese LSCC patients revealed hsa-miR-31, which targets the tumor suppressor *DICER1*, as a potential prognostic biomarker as higher expression was associated with poor patient survival (78).

**Table 2:** Altered miRNAs in LSCC: A detailed list of all the miRNAs used as biomarkers are listed in **Table 2**.

Micro-RNA	Expression in LSCC	Activity	Reference
miR-17/92 clusters - miR-17, miR-18a, miR-19a, miR-19b-1, miR-92a, miR-146b	Upregulated	miR-17 targets 2F1-3, NCOA3, and RBL2 miR-18a targets interferon regulatory factor 2 (IRF2) miR-19a targets the suppressor of cytokine signalling 1 and mediating STAT3 activation miR-19b regulates EGFR signalling pathway by targeting PP2A and BIM miR-92a regulates integrin α5 (ITGα5) miR-146b regulates Estrogen receptor signalling, Nuclear factor-κB signalling and Nucleotide excision repair pathway	(77)
miR-125a-let7e cluster	Down regulated	AT-rich domain 3A (ARID3A)	(77)
mature miR-218	Downregulated	Inhibits EMT by decreasing the IGF-1R level	(78)
miR-210, miR-182, hsa-miR-31	Upregulated	miR-210 likely to target CHRM2 and ADCY9 miR_182 regulates EPAS1, PRKCE, NR3C1, and RHOB has-miR-31 targets the tumour suppressor *DICER1*	(79)
miR-486-5p, miR-30a, and miR-140-3p,	Downregulated	miR-486-5p is involved in targeting PIK3R1 miR-30a targets the SIRT1 3′-UTR miR-140-3p downregulates the expression of ATP8A1	(80)
miR-205	Upregulated	Targets *PHLPP2* and regulates both the AKT/FOXO3a and AKT/mTOR signalling pathways	(80)
miR-139-5p	Downregulated	miR-139-5p targets CXCR4	(81)
miR-106a-5p, miR-93-5p	Upregulated	Regulate metastasis of NSCLC by targeting phosphatase and tensin homolog *(Pten)*, miR-106a-5p targets *ABCA1, and* miR-93-5p is known to inhibit *RB1*	(82)
miR-20a-5p	Upregulated	A member of miR-17-92 cluster acts as an anti-apoptotic agent by targeting *TβRII* in cancer cells	(82)
miR-21a	Upregulated	Regulates programmed cell death 4 (PDCD4) and maspin	(83)
miR-210, miR-708	Upregulated	miR-210 targets autophagy related genes ATG7, LC3-II/LC3-I and Beclin-1 miR-708 targets pro-oncogenic PGE2 signalling	(84)
miR-126, miR-193a-3p, miR-101, miR-15a	Down regulated	miR-126 inhibits ITGA6 miR-193a-3p downregulates ERBB4, targets 3'-UTR of KRAS miR-101 targets zinc finger E-box binding homeobox 1 miR-15a targets and regulates Smad3	(85)
miR-185 and miR-125a-5	Upregulated	miR185 targets SOX9 and regulates Wnt signalling miR-125a-5p targets histone methyltransferase Suv39H1	(85)

The expression and activity of individual miRNA is pointed in this table.

In addition to diagnosis, miRNA profiling has also been used to differentiate LSCC from LUAD. miR-205 and miR-375 can distinguish the two types with 96% accuracy (79). Increased expression of miR-205 was noted in LSCC but was unaltered in LUAD, whereas miR-375 expression was high in LUAD and unchanged in LSCC compared to normal lung tissue (79). Furthermore, increased miR-139-3p and downregulated miR-139-5p in the sputum of LSCC patients may also be potential biomarkers (80). In another study, a panel of three miRNAs (miR-106a-5p, miR-20a-5p, miR-93-5p) were highly upregulated in LSCC patients compared to normal controls and may be potential diagnostic biomarkers (81). In other work miR-375 and miR-10b-5p expression was downregulated in the serum of LSCC patients compared to controls (82). A recent study screened 245 LSCC samples for genome-wide oncogenic miRNAs and compared them with the TCGA dataset (86). 231 of 1,001 miRNAs were associated with copy number alterations from which only 11 were increased in LSCC compared to adenocarcinoma and normal tissues. Three onco-miRNAs (miR-296-5p, miR-324-3p and miR-3928-3p) were specifically associated with poor prognosis (86). Another study showed that in particular, miRNA-21 expression was found in LSCC patients with short survival periods and was associated with poor prognosis (87). A biomarker panel of three miRNA from 15 sputum samples from LSCC patients was also created to detect early stage tumors (88). The study was validated in sputum from an independent set of 67 LSCC patients and 55 healthy controls. They identified over-expression of a three miRNA panel biomarker to detect early stage LSCC. Collectively, miRNAs common to all these studies show that miR-20a-5p, miR-93-5p, miR-106a-5p, miR-146b, miR-205, miR-210, miR-296-5p, miR-324-3p, miR-375, miR-708 and miR-3928-3p may be used as biomarkers and can differentiate LSCC over LUAD. Many of these miRNAs, such as miR-17/92 clusters, miR-20a-5p, miR-93-5p, miR-146b, miR-210 are involved in the regulation of the PTEN/PI3K/AKT/mTOR pathway, an essential regulatory pathway in NSCLC pathogenesis. However, some are also involved in the Wnt/β-catenin pathway such as miR-708, miR-324-3p, in the EMT pathway or are independently involved in LSCC. A detailed list of miRNAs along with their role in the LSCC are listed in **Table 2**.

### Chemotherapeutic, targeted and immunotherapeutic approaches

Despite of understanding the genetic and epigenetic alterations identified in clinical samples of LSCC, there are many limitations in current therapeutic approaches. Chemotherapy, with or without radiotherapy, has remained the main treatment option for patients with LSCC (89–91), and many of these treatments were assessed in mouse models prior to clinical studies. A detailed list of treatment options are documented in **Table 3**. Increased anti-tumor activity of a combination of gemcitabine/cisplatin was established compared to the anti-tumor activity of only gemcitabine or cisplatin in preclinical studies using *in vivo* and *in vitro* approaches (101). LSCC patients undergoing cytotoxic therapy with gemcitabine/cisplatin had greater progression-free survival compared to pemetrexed/cisplatin treated patients (93). A phase III second-line trial revealed that patients treated with pemetrexed had a lower survival rate than those treated with docetaxel (98). Treatment with *nab*-paclitaxel/carboplatin improved the survival rate of LSCC patients compared to paclitaxel/carboplatin with fewer side effects of myalgia, neuropathy, and cytopenia (83). A different study showed that co-administration of necitumumab with gemcitabine/cisplatin improved overall median survival of LSCC patients (94). Bevacizumab (VEGF-A inhibitor) enhanced the anti-tumor activity of erlotinib in xenograft models (95). A study conducted on NSCLC with 25% LSCC patients showed that treatment with ramucirumab (VEGFR2 inhibitor)/docetaxel increased median progression-free survival in LSCC patients compared to placebo/docetaxel (96). Other targets such as FGFR and PI3K-AKT are under investigation.

**Table 3:** Therapeutic drugs for NSCLC: A list of currently available therapeutic options for patients are noted in **Table 3** with its activity in targeting pathway or immune-checkpoint in LSCC.

Name of Drug	Activity	Reference
Pioglitazone	A ligand for peroxisome proliferator-activated receptor-γ –targeting MAPK cascade and TGFβ/SMADs signalling	(92)
Gemcitabine	A pyrimidine nucleoside antimetabolite	(91)
Cisplatin, *cis*-diamminedichloroplatinum (II)	A platinum compound for cancer treatment	(91)
Pemetrexed	A multitargeted antifolate that inhibits enzymes involved in folate metabolism and purine and pyrimidine synthesis	(93)
Paclitaxel	Microtubule polymerization and stabilization in living cells, where it is capable of antagonizing the effects of colchicine and vinca alkaloids	(94)
Carboplatin	A derivative of cisplatin, it binds to DNA, inhibiting replication and transcription and inducing cell death	(94)
Necitumumab	Blocks the interaction between EGFR and its ligands	(95)
Bevacizumab	Inhibits Vascular endothelial growth factor (VEGF)-A	(96)
Erlotinib	Inhibitor of Epidermal growth factor receptor (EGFR)	(96)
Ramucirumab	Inhibits Vascular endothelial growth factor (VEGF)-A	(97)
Nivolumab	Programmed Death-1 (PD-1) Inhibitor	(98)
Pembrolizumab	Programmed Death-1 (PD-1) Inhibitor	(98)
Ipilimumab	A monoclonal antibody that works to activate the immune system by targeting CTLA-4	(99)
Tremelimumab	A monoclonal antibody that works to activate the immune system by targeting CTLA-4	(100)

Immune checkpoint inhibitors (ICIs) such as targeting programmed death-1/programmed death ligand-1 (PD-1/PD-L1) and cytotoxic T-lymphocyte antigen-4 (CTLA-4) blockers have been integrated into standard-of-care regimens for patients with advanced LSCC (97).

LSCCs express PD-L1, enabling escape from immune responses by interacting with PD-1 on T-cells, which suppresses their anti-tumor effects (102). Yu H et. al., assessed the correlation of PD-L1 Expression with tumor mutation burden in early-stage LSCC (100). Nivolumab is a monoclonal antibody (mAb) which targets the PD-1 receptor on T-cells to activate the cells anti-tumor response. A phase II single-arm trial of nivolumab in LSCC patients showed an 11-month overall response rate of 15% (103). A recent phase III trial compared nivolumab to docetaxel as a second-line therapy in 272 advanced LSCC patients after having progressed on platinum-based chemotherapy. Nivolumab treatment increased overall survival by 41% over the docetaxel arm (103). Recent studies established that pembrolizumab (anti-PD-1) alone or combined with platinum-based chemotherapy, is valuable as a standard first-line treatment for LSCC and highly effective in clearing tumors in 20% of cases (104). However, once patients progress on such immune checkpoint inhibitors and chemotherapy, few options are available. A very recent Phase III LUX-Lung 8 study has established that the tyrosine kinase inhibitor (TKI) afatinib can significantly increase overall survival in LSCC patients and may be used as a second line treatment (104). A biomarker driven lung cancer master protocol (Lung-MAP; S1400) was recently performed to address the need of better therapies for LSCC (105). The study evaluated taselisib (targeting PIK3CA alterations), palbociclib (cell cycle gene alterations), AZD4547 (FGFR alterations), rilotumumab (hepatocyte growth factor/scatter factor:MET pathway inhibitor) plus erlotinib (TKI), talazoparib (homologous recombination repair deficiency inhibitor), telisotuzumab vedotin (targeting cMet) and evaluated durvalumab (blocks the interaction of PD-L-1 with the PD-1), and nivolumab (PD-1 Inhibitor) plus ipilimumab (anti-CTLA-4) for anti-PD-1 or anti-PD-L1-naive disease, and durvalumab (anti-PD-L1) plus tremelimumab (another anti-CTLA-4) for anti-PD-1 or anti-PD-L1 relapsed cancers. Seven % of patients responded to targeted therapy and 16·8% patients responded to anti-PD-1 or anti-PD-L1 therapy for immunotherapy-naive disease (105). A recent study has established that dual immunotherapy along with chemotherapy enhances clinical benefit such as longer overall as well as progression free survival. This study used nivolumab plus ipilimumab along with platinum doublet chemotherapy and chemotherapy alone. A significantly improved overall survival was noted compared to chemotherapy alone and also had a favorable risk–benefit profile (106). Another report showed that nivolumab plus ipilimumab with two cycles of chemotherapy has a long-term, durable efficacy up to 3-year minimum follow-up and survival benefit in LSCC patients with brain metastases (107).

Compared to LUAD, there has always been a lack of effective targeted treatments for LSCC and limited progress has been made in the systemic treatment of advanced disease. Despite of the several available options for the treatment of NSCLC, few of these treatments are specific to LSCC and therefore new therapies are urgently required. Importantly, most of these current therapies for NSCLC were developed in studies that used immunocompromised animals. The development of robust, short-term mouse models of LSCC in immunocompetent animals and that recapitulate human disease features by combining CS with carcinogens such as NTCU, is essential for increasing the understanding of disease pathogenesis as well as improving the efficacy of current therapies and informing novel therapeutic strategies for LSCC.

### Murine models of LSCC

Pre-clinical animal models are crucial in cancer research and are valuable tools for understanding the underlying mechanisms involved in tumor initiation and progression. They also provide a platform to test the safety and efficacy of novel treatments. While an increasing number of naturally occurring cancers in animals, notably dogs, are being explored for these purposes (99, 108, 109), mouse models remain the mainstay of pre-clinical studies.

#### Chemical-induced mouse models

Chemical-induced mouse models can also be used to model human disease since they drive human cancers. In one of the earliest studies, BC3F1 (a cross between C57BL and C3H inbred strains) and DBA/2 mice were intratracheally administered 3-methylcholanthrene (MCA, 0.5mg) once a week for 6 and 4 weeks, respectively, and rested for up to 7 months (110). After 24 weeks, 86% of BC3F1 mice LSCC, however, only 3/50 DBA/2 mice developed tumors of squamous cell origin after 7 months (110). Intratracheal instillation of benzo[a]pyrene (BAP) with charcoal powder once a week for 8 weeks induced moderately to highly keratinized LSCC in C57BL/6 mice after 40 weeks (111). However, these models have not been used subsequently. In a different study, female Swiss mice were administered 40mM of N-nitroso compounds such as nitrosoalkylureas, nitrosoalkylcarbamates, and chlorinated nitrosotrialkylureas topically in the subscapular region, twice a week for 50 weeks. Eleven/20 mice administered nitroso-tris-(2-chloroethyl)urea (NTCU) developed LSCC after 110 weeks (112).

These NTCU model although exciting was challenging to reproduce until Wang et al., further developed the NTCU model treating eight inbred mouse strains (129/svJ, AKR/J, BALB/cJ, C57BL/6J, FVB/J, SWR/J, A/J,NIH Swiss) with NTCU using the same protocol and examined lung histology after 8 months to discover the NTCU was only able to indcuse LSCC in 5 of the 8 strains. The SWR/J, NIH Swiss, A/J, BALB/cJ, and FVB/J all developed tumors, where A/J, NIH Swiss and SWR/J mice were most susceptible to NTCU skin treatment, FVB/J and BALB/cJ mice had intermediate susceptibility, and AKR/J, 129/svJ and C57BL/6J mice were resistant (113). Most susceptible strains of mice progressively develop hyperplasia, metaplasia and invasive LSCC (75-100% incidence), whereas mice with intermediate susceptibility have ~45% incidence of invasive LSCC. Resistant strains had no evidence of invasive LSCC, although they had hyperplasia, metaplasia and carcinoma *in situ*. A more recent study though sought to limit some of the toxicity associated with NTCU has shown that A/J mice administered with NTCU (13 mM) for 2, 4, and 8 weeks, topically in the subscapular region and sacrificed 18 weeks had 25, 54 and 71% LSCC incidence, respectively, with LSCC originating in deltaNp63^+^CD44v^+^ club (Clara) cells (114). Whereas a study in FVB mice with topically administered NTCU (4, 8, or 40 mM) for 32 weeks, demonstrated that 4 mM and 8 mM NTCU although were well tolerated only induced flat atypia whereas 40mM lead to dysplasia and LSCC (115). Further, also in FVB mice, 20 mM NTCU twice a week for up to 32 weeks did not show any dysplastic changes until the 25^th^ week and bronchial dysplasia and LSCC occurred after 32 weeks (116).

A recent study used transcriptome (RNA) sequencing (RNA-Seq) to profile bronchial airway gene expression in an NTCU-induced LSCC mouse model (117). Swiss mice were treated topically with 40 mM of NTCU (twice a week) for 24 weeks to induce early LSCC lesions. They found activation of oncogenic PI3K and Myc pathways in bronchial epithelial cells of mice with preneoplastic changes. The authors also showed that the expression of mouse microRNA (miRNA)-449c-5p (mmu-miR-449c-5p) was suppressed in cells with upregulated *myc* expression (117). Another recent study assessed and compared the effects of different topical doses of NTCU (20, 30 and 40 mM) in both female and male NIH Swiss, Black Swiss and FVB mice. NIH swiss mice had a higher LSCC incidence and lower mortality with 30mM of NTCU (118). One recent study has incorporated tobacco smoke with NTCU to enhance the efficacy of NTCU to develop pre-malignant lesions of LSCC. This study used a whole-body smoke system to expose A/J mice to CS for 3-6 weeks following 4-5 weeks of 20mM NTCU administration (twice a week) and concluded that NTCU combined with CS exposure leads to the development of LSCC in a shorter time period and would be more relevant to human disease (119). Although these models are more relevant to human disease compared to xenograft and transgenic models, they have their own limitations such as prolonged cytotoxic treatment leading to ethical challenges. Moreover, the recent combination of NTCU with CS exposure used whole-body smoke exposure systems which is more comparable to passive rather that active smoking. Although induce early neoplastic changes are induced, human LSCC occurs in active chronic smokers. Thus, nose-only/inhaled CS exposure combined with NTCU would give more relevant neoplastic changes in murine models.

#### Carcinogen-induced models used in chemoprevention studies

Carcinogen-induced murine SCC models have been employed in several chemoprevention studies. One of the earliest assessed the efficacy of anti-tumor B (ATB), which is also known as Zeng Sheng Ping (a Chinese herbal mixture), in treating SCCs in A/J mice that were induced by topical administration of NTCU (40 mM) twice a week for 22 weeks. Mice were treated with AIN76APurified Diet (normal control mouse chow) along with 250 g/kg ATB. ATB reduced the development of lung SCC (3.1-fold; *p*<0.05) but efficacy was not tested in human SCC patients (92). Another study assessed the anti-cancer activity of pioglitazone, a ligand for peroxisome proliferator-activated receptor-γ. The skin of female NIH Swiss mice was painted with of NTCU (30 mM/L) twice a week, with 3-day intervals for 32 weeks, and pioglitazone (15 mg/kg body weight) treatment was administered by oral gavage beginning 8 weeks after the first NTCU treatment. Treatment inhibited the progression of SCC by 35% (*p*<0.05) (120). The chemopreventive effect of Korea white ginseng (KWG) was assessed in NTCU-induced SCC in female Swiss mice. KWG was given orally with 30 mM/L NTCU administered topically twice a week with a 3.5-day interval for 24 weeks. KWG treatment blocked the progression of SCC (121). In another study, female A/J mice were given NTCU (0.5 mmol/L) and lipopolysaccharide (LPS, 4 µg) intranasally once a week for 26 weeks. Mice were fed a diet containing diindolylmethane (DIM, 10 µmol/g), which is one of the breakdown products of indole-3-carbinol, in AIN-93G/M powder (high protein and fat) throughout. LPS has pro-carcinogenic activity since mice treated with NTCU and LPS had 9-fold increases in bronchiolar SCCs, the chemopreventive capacity of DIM was demonstrated with a 2-fold reduction in SCC (122). Green tea provided as drinking water for A/J mice 2 weeks after the first NTCU treatment (40mM, twice a week for 32 weeks) reduced the development of SCC to 5/9 mice (56%) compared to 90% observed in NTCU only treated controls (123). In another study, the efficacy of pomegranate fruit extract (0.2% in drinking water throughout) as a chemoprevention agent in female A/J mice administered BAP or NTCU (140 and 240 days, respectively) was assessed. Treatment reduced tumor incidence by 53.9% and 65.9%, respectively, which was associated with down-regulated MAPKs, NF-κB, PI3K, and mTOR signalling networks, compared to untreated controls (124). The chemopreventive effect of dietary vitamin D was assessed in NTCU-treated SWR/J mice. Dysplastic changes were increased in NTCU mice on a low vitamin D diet compared to those on a normal/high vitamin D diet (125).

Recently C57BL/6 mice were used to examine the role of *Rad52*, which is involved in DNA repair mechanisms. In this study, NTCU (30 mM/L) was administered topically twice weekly with a 3.5-day interval for 38 weeks to *Rad52*-deficient (^-/-^) mice. Deletion of *Rad52* increased tumor cell death and reduced growth compared to wild-type (WT) mice that appeared to be due to enhanced *Rad52* ^-/-^ NK and CD8^+^ T-cell effector functions (126).

#### Transgenic models

Mutations in serine/threonine kinase-11, also called *Lkb1*, are implicated in epithelial cancers. A *Kras-*mutant mouse model was used to analyse the role of *Lkb1.* Simultaneous activation of *Kras* ^G12D^ (*Kras*) and inactivation of *Lkb1* led to LSCC and also other types of LC (127). However, inactivated *Lkb1* did not lead to LSCC if *Kras* ^G12D^ was not activated. Nevertheless, *Kras* mutations are generally not observed in human LSCC (128). Inactivation of *Lkb1* alone is insufficient for LSCC development but an incidence of 100% LSCC was induced in mice that lost *Lkb1* and *Pten* after 40-50-weeks of latency (129). LSCC markers such as *Sox2*, were over-expressed with lentiviral delivery of *Sox2* to the lungs of *Lkb1^-/-^* mice, which promotes LSCC by activating STAT and mTOR pathways. These mice express orthologues of human LSCC biomarkers, such as cytokeratin-5, 14, and p63 (130). A recent study defined the significance of *Ikkα* in LSCC development in mice. Kinase-dead IKKα knock-in (*Ikkα^K44A/K44A^, Ikkα^KA/KA^*) mice on an FVB background were generated to study the levels of *Ikkα^KA/KA^* expression at different stages and its relationship to LSCC development. These mice started developing spontaneous lung tumors after 3 months of age. IKKα levels were high in newborn WT compared to *Ikkα^KA/KA^* mice. IKKα levels were lowest in 4 month old *Ikkα^KA/KA^* mice. The reduction in IKKα levels could contribute to LSCC (131). Recent studies have established that *SOX2* overexpression in tracheobronchial basal cells combined with *CDKN2AB* and *PTEN* loss results in LSCC (132). This study also confirmed that although overexpression of *FGFR1* transforms *PTEN*- and *CDKN2AB*-deficient tracheobronchial basal cells at a higher frequency, the tumors formed were heterogeneous with occasional squamous differentiation. It is also established that NKX2–1 suppresses SOX2-driven squamous tumorigenesis (133).

#### Xenograft models

Patient-derived xenografts (PDXs) have the potential to replicate the histopathological features, heterogeneity and gene expression pattern of the original tumor (134, 135). LSCCs have increased engraftment compared to other NSCLCs (135). Clinically relevant models of LSCC have been attempted by implanting human LSCCs from surgical resections and biopsy specimens subcutaneously into the flanks of immune-deficient NOD.SCID*^prkdc^Il2rγ* ^−/−^ (NSG) mice, which resulted in 52% and 33% tumor take up, respectively (136). Engrafted LSCCs maintained the patient’s tumor phenotype for at least four passages. This model was used to show that *FGFR1* mRNA detection is a better predictor of response to FGFR inhibitors than *FGFR1* gene amplification. Sixty-two PDX LSCC models were developed that showed similar gene expression profiles, pY-proteome landscapes (profile to compare similarities between PDX models) and DNA methylation patterns as human tumors (137). Another study was only able to establish 35 PDX models out of 100 human tumors because of contamination by lymphomagenesis (138). In a separate study, 21 PDX models were established from 36 patients, with these xenograft tissues analyzed and determined to retain LSCC characteristics, including CK5/6, p63 and p40 expression (139). In the most recent study, 18 PDXs were established from 37 surgical specimens with 16/18 passaged to 2^nd^-3^rd^ generations and were seen to develop LSCC. Histological markers, such as p53, p63, cytokeratin5/6, and E-cadherin, as well as response to cisplatin, were retained in the mouse tumors. Altered expression of Ki-67, long non-coding RNA and mRNA were observed in 3^rd^-generation xenografts (140).

Collectively, these studies further the understanding of human LSCC development and provide information for non-immune based therapies. However, there are limitations including the high mortality rate due to long-term cytotoxic treatment, low success rate of engraftment and unsuitability for studying anti-tumor immune responses, as the mice are immunodeficient. Thus, it is crucial to develop LSCC models in immunocompetent mice with reduced mortality rate and cytotoxic effects. Short term cytotoxic insults along with tumorigenic driving factors such as CS and CS-induced COPD in immunocompetent mice could provide a more robust murine model to study the pathogenesis and establish the diagnostic biomarkers of the disease.

#### Syngeneic mouse model

The lack of animal models that reflect human disease to assess the safety and efficacy of drugs and to explore the underlying molecular mechanisms is one of the major impediments in LSCC research. Syngeneic models are allografts immortalized from mouse cancer cell lines, which are then engrafted back into the same inbred immunocompetent mouse strain. Valencia et al., 2022 generated and characterized two syngeneic lung SCC cell lines i.e., UN-SCC679 and UN-SCC680 derived from NTCU carcinogen treated A/J mice. They observed similar genetic and transcriptomic patterns that may correspond to the classic LUSC human subtype. In addition, they compared the immune landscape generated by both tumor cells lines *in vivo* and assessed their response to immune checkpoint inhibition. The differences between the two cell lines are a good model of the broad heterogeneity of human SCC. Studies of the metastatic potential of these models revealed that both cell lines represent the organotropism of LUSC in humans, i.e. affinity for the brain, bones, liver and adrenal glands (141).

Azpilikueta et al., 2016 used the transplantable mouse lung SCC UN-SCC680AJ cell line to test the efficacy of immunotherapy (anti–PD-1/PD-L1 and anti-CD137 mAbs). In syngeneic mice, the tumors derived from UN-SCC680AJ cells were amenable to curative treatment with anti–PD-1, anti–PD-L1, or anti-CD137 immunostimulatory mAbs. Single-agent therapies lost curative efficacy when treatment was started beyond day +17, whereas a combination of anti–PD-1 plus anti-CD137 achieved complete rejection of the tumors. Tumor cells expressed weak baseline PD-L1 on the plasma membrane, but this could be readily induced by interferon-γ. Combined treatment efficacy required CD8 T cells and induced leukocyte infiltration in which T lymphocytes co-expressing CD137 and PD-1 were prominent. These promising results advocate the use of combined anti–PD-1/PD-L1 plus anti-CD137 mAb immunotherapy for the treatment of squamous non–small cell lung cancer in the clinical setting (142). Thus, these cell lines recapitulate the complexity of the human disease, and could be promising tools for lung SCC research.

#### Organoid models

These are the models that recapitulate the complex genetic profile of patients and could be another promising tool in lung SCC research. Hai et al., 2020 used CRISPR genome editing to delete multiple tumor suppressors in lung organoids derived from Cre-dependent SOX2 knock-in mice. They investigated both the therapeutic efficacy and immunological effects accompanying combination PD-1 blockade and WEE1 inhibition in mouse models and LSCC patient-derived cell lines. They show that multiplex gene editing of mouse lung organoids using the CRISPR-Cas9 system is an efficient and rapid means to generate LSCCs that closely mimic the human disease at the genomic and phenotypic level. Using this genetically defined mouse model and three-dimensional tumoroid culture system, they show that WEE1 inhibition induces DNA damage that primes for endogenous type I interferon responses and antigen presentation system in primary LSCC tumor cells. These events promote cytotoxic T cell-mediated clearance of tumor cells and reduce the accumulation of tumor-infiltrating neutrophils. Beneficial immunological features of WEE1 inhibition are further enhanced by the addition of anti-PD-1 therapy. They developed a mouse model to study a combination approach for immune checkpoint blockade with DNA damage-inducing therapies in the treatment of LSCC (143).

#### Genetic alterations involved in LSCC identified in various in murine models

Tumor genomic organisation differs between the subtypes of NSCLC. Profiling the genomic structure for each subtype of LC is essential to advance therapeutic treatments. An early study examined the effects of a resected human tumor xenograft (T1 lesion contained within the lung without spread to main bronchi with a moderately differentiated LSCC phenotype) in NSG mice (144). RNA was extracted from both CD133^−^/EpCAM^+^ and CD133^+^/EpCAM^+^ subpopulations of LSCC samples and whole transcriptome libraries were sequenced on the SOLiD platform. Both CD133^−^ and CD133^+^ subpopulations involved gene copy number alterations. The expression of 992 genes was assessed. 124 moderately expressed isoforms (80 genes) were selected of which 24 genes were down-regulated and 19 were over-expressed in CD133^+^ versus CD133^-^ cells. A signature of 22 genes encoding cell surface proteins was highly expressed in CD133^+^ cells. Results were compared to the Cancer Genome Atlas Program (TCGA) transcriptome data of 221 LSCCs. Most of the 22 cell surface genes were robustly expressed in all TCGA samples. Since the majority of these alterations are common between humans and mice, they can be readily assessed for potential targeted therapy.

Recently, LSCCs from NIH Swiss mice 32-weeks after NTCU challenge were subjected to whole exome and single cell (sc)RNA-seq. Sixteen resected tumors from mice and eight normal lungs were sequenced to a read depth of 129X (98-165X) and 137X (112-179X), respectively. A total of 5,664 somatic coding mutations were identified, including missense, nonsense, silent mutations, and small insertions and deletions. Fifty-nine genes were recurrently mutated the most frequently mutated were: *Muc4, Prg4, Igf2r, Ctsll3, Dlgap1, Hspa9, Armcx3, Cdk1, Pcdhb15, Fus, Gga1, Il2rb, Kmt2d (Mll2), Mapk6, Myh1, Ncoa3 (Src3), Obscn, Runx2, Zmynd8, Ido1, Nkain2, Pyy, Stil, Tcl1b4, Tfeb* and *Trpv1*. Murine LSCC mutations were compared to 191 human LSCC documented in the catalogue of somatic mutations in cancer (COSMIC) database (145), and of the 47 genes shared between mouse and human LSCC the most common were: *KMT2D* (*MLL2*), *MYH1*, *OBSCN*, *ZEB2*, *BRAF*, *IGF2R*, *FLT1*, *HIVEP3*, *PRG4*, *ABCA1*, *ATR*, *DACH2*, *ABCB4*, *DST* and *MUC4.* However, approximately 20% of recurrent mutations were not shared between human and mouse LSCC. scRNA-Seq of two mouse tumors revealed different sets of mutations (146). In one tumor a clonal mutation (R45P) in *Igfbp7* was identified along with a mutation (R2457S) in *Igf2r* and others in *Ahctf1*, *Notch4*, *Ncoa3*, and *Nfe2l2.* The other tumor had a *Trp53* somatic missense mutation along with mutations in the driver genes *Myh9*, *Kmt2d* and *Keap1*. Although the genomic alterations were documented, the controls were from different mice making it a limitation in this study. Taken together, these studies define a set of altered genes (such as *Zeb2*, *Braf*, *Igf2r, Flt1, Atr, Muc4, Ncoa3 Src3)* that occur in mouse models and are also present in the TCGA of LSCC and may be involved in pathogenesis. However, a wide range of alterations are not shared between the murine models and with TCGA. Moreover, these studies do not cover a wide range of other ‘omics (including proteomics, metabolomics and metagenomics) and epigenetic analysis. These limitations indicate the need for more accurate mouse models that better recapitulate the hallmark features of human LSCC. **Table 4** determines a list of exclusive mutations found in murine models and certain common mutated genes between murine and human LSCC, indicating the dire need of a better murine model to study genetics of LSCC and also would include proteomics, metabolomics and metagenomics and epigenetic analysis.

**Table 4:** Profiles of recurrently mutated LSCC genes in NTCU induced mouse: A list of 59 genes that were identified in NTCU induced mouse model are documented in **Table 4** (146).

LSCC genes exclusively in NTCU induced murine model	Frequently Mutated genes common in murine models and human LSCC	Less Frequently Mutated genes common in murine models and human LSCC	Least Frequently mutated genes common in murine models and human LSCC
**Ctsii3**	**KMT2D**	**ABCB4**	**ZMYND8**
**Dlgap1**	**MYH1**	**DST**	**FOXP1**
**Cdk1**	**OBSCN**	**MUC4**	**HSPA9**
**Pcdhb15**	**ZEB2**	**DLGAP1**	**NKAIN2**
**Gga1**	**BRAF**	**GAK**	**RUNX2**
**ll23b**	**IGF2R**	**JAK3**	**TFEB**
**Pyy**	**FLT1**	**BUB1B**	**TNFRSF13B**
**Bcl3**	**HIVEP3**	**RET**	**CCDC6**
**E0030030106Rik**	**PRG4**	**SFRP4**	**FUS**
**Xaf1**	**ABCA1**	**FANCA**	**GIP**
**Adgre5**	**ATR**	**KDM3A**	**IDO1**
**Hspb1**	**DACH2**	**MAPK6**	**SMO**
	**STIL**	**NCOA3**	**TRPV1**
		**TCL1B**	**ARMCX3**
			**CD55**

This also included the cosmic data base (http://cancer.sanger.ac.uk/cosmic) where the murine mutations were compared for human LSCC relevance.

## Conclusions

The global burden of LSCC is substantial and continues to increase, primarily due to the advanced stage of the disease at the time of diagnosis. CS exposure is the primary risk factor and creates a wide field of injury in the epithelium of the lower airways. Although several biomarkers are established for NSCLC, differentiating LSCC from the other NSCLCs remains a major challenge. There are only poorly effective therapies for LSCC, which are typically applied late due to late diagnosis that further reduces efficacy. Although many studies have been performed on resected human samples, there are multiple limitations. Longitudinal analysis of the developmental and prognostic stages of LSCC is not possible to study in humans. Thus, the use of murine models is important to define the early induction mechanism of LSCC and identify early biomarkers and therapeutic targets and develop and test new therapies that can be progressed into improved treatments.

Current established murine models of LSCC use chemical induction using NTCU, human xenografts and genetically-modified mice. The use of these models has contributed to elucidating the molecular alterations. However, they do have challenges such as high mortality from long-term cytotoxic treatment. They also have limitations where xenograft models do not recapitulate the hallmark features of human LSCC since immunocompromised mice are required, which reduces their applicability in elucidating the pathogenesis of neoplastic changes. Most current therapies were developed in immunocompromised animals. Although chemically-induced mice have challenges, they seem to better recapitulate the features of human LSCC. These models can be improved by combining with low-doses of carcinogens with CS, which is the primary cause of LSCCs in humans, in wild-type mice. This would provide an improved method of inducing LSCC in wild-type/non-genetically-modified mice that better replicate the changes in human LSCC. Using more resistant strains such as C57BL/6 mice to induce LSCC would lead to improved models to study the development and progression of LSCC. Although it is established that C57BL/6 mice are resistant to NTCU, which is widely used to induce LSCC in mice, they are susceptible to CS-induced COPD. Thus, C57BL/6 mice when challenged with both NTCU and CS, may develop LSCC.

The expression of some miRNAs, such as miR-106a-5p, miR-20a-5p and miR-93-5p, is increased in LSCC patients and are often useful diagnostic biomarkers for LSCC. These biomarkers are identified only in advanced stage of the disease. Murine models with low ethical challenges (in terms of time taken for approval, multiple rounds of amendments, strict regulations involved in use of carcinogen etc) that accurately replicates the hallmark features of LSCC would also enable the identification of early biomarkers of the disease.

Despite new therapies being tested for NSCLC, this has not yet improved the survival of LSCC patients. This can only be brought about by increasing the understanding of the disease-causing mechanisms and identifying new therapeutic targets particularly those that are altered early in tumorigenesis and the development and testing of new treatments. This can be achieved by developing murine models of LSCC that address and/or eliminate current limitations to identify and enable rigorous interrogation of underlying genetic and epigenetic changes, including miRNAs.

## Author contributions

PS: Conceptualization, Writing – original draft, Writing – review & editing. CD: Conceptualization, Supervision, Validation, Writing – review & editing. KP: Visualization, Writing – review & editing. SP: Visualization, Writing – review & editing. VC: Visualization, Writing – review & editing. RK: Supervision, Visualization, Writing – review & editing. JH: Supervision, Visualization, Writing – review & editing. KD: Visualization, Writing – review & editing. AI: Visualization, Writing – review & editing. FN: Visualization, Writing – review & editing. HB-O: Investigation, Visualization, Writing – review & editing. SM: Visualization, Writing – review & editing. GC: Visualization, Writing – review & editing. JL: Visualization, Writing – review & editing. PH: Conceptualization, Funding acquisition, Resources, Supervision, Visualization, Writing – review & editing.

## Glossary

**Table:**

AKT	Protein kinase B
ALK	Anaplastic Lymphoma Kinase
ARID3A	AT-rich domain 3A
ATB	Anti-tumor B
BAP	Benzo[a]pyrene
CHRNA	Cholinergic nicotinic receptor locus
COPD	Chronic Obstructive Pulmonary Disease
CS	Cigarette Smoke
CTA	Cancer/Testis Antigens
DDR2	Discoidin Domain Receptor Tyrosine Kinase 2
EGFR	Epidermal growth factor receptor
EPHA2	Ephrin type-A receptor 2
FAM13A	Family with sequence similarity 13, member A
FGFR1	Fibroblast Growth Factor Receptor
*IGF1R*	Insulin-like growth factor-1 receptor
LAC	Adenocarcinoma
LC	Lung Cancer
LOH	Loss of heterozygosity
miRNA	Micro RNA
NF-κB	Nuclear factor-kappaB
*NOTCH1*	Notch homolog-1, translocation-associated
*NRF2*	Nuclear factor erythroid-2-related factor*-*2
NTCU	Nitroso-tris-(2-chloroethyl)urea
PAH	Polycyclic aromatic hydrocarbons
PDL-1	Programmed death ligand receptor 1
PD-1	Programmed death receptor 1
PDGFRA	Platelet-derived growth factor receptor A
PDX	Patient Derived Xenograft
PI3K/PIK3CA	Phosphatidylinositol-4,5-bisphosphate 3-kinase
PTEN	Phosphatase and tensin homolog
RNA-Seq	Transcriptome sequencing
LSCC	Squamous Cell Carcinoma
SCLC	Small Cell Lung Carcinoma
SOX2	SRY (sex determining region Y)-box 2
TNF-α	Tumour Necrosis Factor alpha
VEGF	Vascular endothelial growth factor.
